# Standard operating procedure for the analysis of major ions in hydrothermal fluids by ion chromatography

**DOI:** 10.12688/openreseurope.15605.2

**Published:** 2024-05-28

**Authors:** Monica Correggia, Luciano Di Iorio, Alessia Benedicta Bastianoni, Mustafa Yucel, Angelina Cordone, Donato Giovannelli

**Affiliations:** 1 Department of Biology, University of Naples Federico II, Naples, Italy; 2 Institute of Marine Sciences, Middle East Technical University, Mersin, Turkey; 3 Marine Chemistry & Geochemistry Department, Woods Hole Oceanographic Institution, Woods Hole, USA; 4 Earth-Life Science Institute, ELSI, Tokyo Institute of Technology, Ookayama, Japan; 5 Department of Marine and Coastal Science, Rutgers University, New Brunswick, USA; 6 Istituto per le Risorse Biologiche e Biotecnologiche Marine, IRBIM, National Research Council, Italy, Ancona, Italy

**Keywords:** major ions, hydrothermal fluids, ion chromatography, Standard Operating Procedure, aqueous geochemistry

## Abstract

This standard operating procedure (SOP) describes an ion chromatography (IC) procedure for the major cations and anions in hydrothermal fluids. Hydrothermal fluids are aqueous solutions with a wide range of temperature, salinity, pH and ion species that can be used by microbial metabolism as electron donors and electron acceptors. Due to the high variability of the environmental physical-chemical parameters in these samples, we have developed this protocol taking into account the special features of the matrices analyzed. An Eco IC Metrohm system equipped with a conductivity detector was used. Calibration curves are linear in the 0.1 to 10 mg/L concentration range for cations Ca ^2+^, Na ^+^, K ^+^, Mg ^2+^, NH _4_^+^ and anions Cl ^-^, Br ^-^, NO _3_^-^, NO _2_^-^, SO _4_^2-^ , HPO _4_^2-^.

## Plain Language summary

This manuscript details the standard operating procedure used for the determination of major cations and anions in hydrothermal fluids, which are characterized by a high variability of physico-chemical parameters. Due to the high variability of our samples, we customized the method to compensate for the variability on the physico-chemical parameters. The results are useful to interpret the geochemical processes and for understanding how these processes influence microbial diversity in geothermal systems.

## Introduction

This method was developed in the Giovannelli Lab at the Department of Biology of the University of Naples Federico II for the sequential measurement of major cations (Ca ^2+^, Na ^+^, K ^+^, Mg ^2+^, NH _4_^+^) and anions (Cl ^-^, Br ^-^, NO _3_^-^, NO _2_^-^, SO _4_^2-^ and HPO _4_^2-^) in hydrothermal fluids using ion chromatography. This analysis can provide different information, in fact it is well known that these elements are required for the nutrition and metabolism of microbes, in particular for growth, oxidative metabolism and active transport. Some of these species can be used by microorganisms for redox reactions ( Hay Mele *et al.*, 2023; Moore *et al.*, 2017), while others can inform on the nature and origins of fluids under investigation ( Giggenbach, 1988). Microbial diversity is primarily driven by physical-chemical conditions, such as temperature, pH, redox state, and availability of diverse electron donors and electron acceptors ( Delgado-Baquerizo *et al.*, 2018; Merino *et al.*, 2019). For example, chemolithotrophic bacteria can obtain the energy required for their growth from oxidation of inorganic compounds like nitrate, sulfide or ammonium ( Beller *et al.*, 2006; Bock *et al.*, 1990). Chemolithoautotrophic ecosystems support a set of metabolic processes tightly connected to deep subsurface geological and geochemical parameters ( Fullerton *et al.*, 2021; Rogers *et al.*, 2023). Macroelements can be correlated with prokaryotic microbes to understand whether geochemical parameters affect the composition of microbial communities ( Liu *et al.*, 2018). Moreover, tectonic activity can act as a source of substrates for microbial life ( Falkowski *et al.*, 2008; Giovannelli *et al.*, 2022), indeed tectonically controlled geochemistry of hydrothermal fluids resulting from water-rock interaction are correlated with bacterial community composition ( Fullerton *et al.*, 2021). Therefore, the abundance of ionic species in fluids can expand our knowledge of geochemical processes that could have important implications for microbial communities.

Deeply-sourced seeps, encompassing a wide variety of diverse features that include fumarole, mofettes, acid-sulfate springs, deep chloride springs, steam heated waters and alkaline soda springs ( Giovannelli *et al.*, 2022), show extremely diverse ionic compositions accompanied by wide variations in temperature, pH, salinity and redox conditions. Our dataset routinely includes samples with pH varying between 0.5 to 11.2, temperatures ranging from 2 °C to 375 °C and salinity from 0 % to 35 %. Here we describe the standard operating procedure (SOP) developed to routinely analyze such a wide range of different fluid compositions.

## Methods

Samples are injected through a series of ion exchange columns until they reach the conductivity detector. The first column effectively protects the analytical column from contaminants that may be present in the samples, extend the life of the analytical column and has no effect on chromatographic separation performance. The analytical column separates ions according to their affinity with the selective ion-exchange column using high-purity mobile phase. For anionic chromatography, the suppressor reduces the background conductivity of the eluent and enhances the conductivity of the analytes. Finally, analytes are identified according to their retention time and the quantification is carried out through certified standards. We developed this protocol to work with a single ion chromatograph on which we change the column to analyze cations and anions in different runs, however the described SOP can be used only for cations or anions, or to analyze them in parallel with two ion chromatographs that are setup to run simultaneously.

As previously mentioned, hydrothermal fluids are aqueous solutions with a wide range of temperature, salinity and pH that requires specific adjustments compared to the routine analysis of drinking water or seawater for which these parameters vary less on a per sample basis. Given the presence of a conductivity detector and the ionic exchange that takes place in the column, care must be taken to keep the sample conductivity near or under 600 μS/cm. This extends column lifetime and increases the signal to noise ratio. Additionally, the presence of some ionic species in excess of others in the sample might interfere with peak detection. For example, chloride and sodium can hide the peaks of nitrite and ammonium, and the presence of high concentrations of organics can sometimes overprint the entire chromatogram significantly, increasing the detection limits for the specific sample. These issues can generally be resolved using sample pretreatment such as offline silver ion exchange columns for chloride, or solid phase extraction C18 columns to selectively remove organics. Additionally, ions exist in solution in different forms depending on the pH of the solution, so the pH of the samples may affect ion exchange within the column. This can be avoided by keeping the ratio between the volume of the sample and the volume of eluent very small (usually 1:1000) and pre-diluting the sample (at least 1:10 dilution for our specific case) before injection and always below 600 μS/cm.

### Standard operating practice for deeply-sourced seeps fluids

The method was developed using a Metrohm ECO-IC ion chromatograph (1.925.0020) equipped with a conductivity detector (1.850.9010). In order to reduce the total dissolved solids, all samples are filtered in the field through a 0.22 μm filter. The samples are then stored at 4 °C in the dark until analysis. Once in the lab, samples are diluted to reduce the specific conductivity to below 600 μS/cm (measured in the field using a HANNA instrument multiprobe, HI98194). All dilutions are made with type I water (18 MΩ/cm), which is also used as blank. Anions are run using a 3.2 mM Na _2_CO _3_ + 1 mM NaHCO _3_ mobile phase with a Metrosep A Supp 5 column (Metrohm) equipped with a 0.15 M ortho-phosphoric acid suppressor with a flow of 0.7 ml/min for 30 min. Cations are run using 2.5 mM HNO _3_ + 0.5 mM (COOH) _2_∙2H _2_O mobile phase with Metrosep C4 column (Metrohm) with a flow of 0.9 ml/min for 35 min. Data acquisition and analysis is carried out through MagIC Net 3.3 software provided with the instrument. Calibration curves are designed using certified external standards (CPAchem) for each of the anions and cations analyzed. Calibration curves for the ion species of interest are run in the range of 0.1 and 10 ppm with r ^2^ ≥ 0.999 at least once a month, and a 1 ppm multistandard containing all the ions of interest is run every 10 samples to check for recovery and peak drift.

### Apparatus and equipment

- Eco IC ion chromatography (1.925.0020 Metrohm, Switzerland)
- 863 Compact IC Autosampler (1.863.0010 Metrohm, Switzerland)
- Metrosep A Supp 5 column (6.1006.530 Metrohm, Switzerland)
- Metrosep C4 column (6.1050.430 Metrohm, Switzerland)
- Metrosep A Supp 5 Guard/4.0 (6.1006.500 Metrohm, Switzerland)
- Metrosep C4 Guard/4.0 (6.1050.500 Metrohm, Switzerland)
- Vent Filter MPK01 (TANKMPK01 Merck, USA)
- MagIC Net 3.3 software (Metrohm, Switzerland)
- Analytical balance capable of 0.0001 g sensitivity (MS105DU Mettler Toledo, USA)
- Class A volumetric flasks (VFL3-010-002, VFL3-100-002 Labbox)
- Pipettes for reagent and standard preparation
- PP syringe filters 0.22 μm (TS-900-063 Perlabo)

### Reagents and standards

***Stock eluent solution***

- Anions eluent stock solution. A fresh batch of eluent solution is prepared before each run. Dissolve 0.678 g of Na _2_CO _3_ extra pure (99 % grade) and 0.168 g of NaHCO _3_ extra pure (99 % grade) in 80 ml type I water in a 100 ml volumetric flask. When dissolution is complete, bring up to 100 ml with type I water. Final concentration: 3.2 mM Na _2_CO _3_ + 1 mM NaHCO _3_.
- Cations eluent stock solution. A fresh batch of eluent solution is prepared before each run. Dissolve 0.63 g of (COOH) _2_∙2H _2_O extra pure (99 % grade) in 80 ml type I water in a 100 ml volumetric flask and add 1.67 ml of 67 % HNO _3_. When dissolution is complete, bring up to 100 ml with type I water. Final concentration: 2.5 mM HNO _3_ + 0.5 mM (COOH) _2_∙2H _2_O.

***Working eluent solution***

- Anions eluent working solution. Take 50 ml of eluent concentrate into a 1 L volumetric flask and transfer into a glass bottle. Before use, degas the solution using vacuum until no more bubbles can be detected.
- Cations eluent working solution. Take 10 ml of eluent concentrate into a 1 L volumetric flask and transfer into a glass bottle. Before use, degas the solution with a vacuum until no more bubbles can be detected.

***Suppressor regenerant solution***

To reduce the background conductivity of the eluent and enhance the conductivity of the analytes, we use ortho-phosphoric acid (high purity). Pipette 5.13 ml of 85% H _3_PO _4_ in 400 ml type I water in a 500 ml volumetric flask. When dissolution is complete, bring up to 500 ml with type I water. Final concentration 0.15 M.

***Washing solution***

To reduce the organic contamination of the injection system and column we use an ethanol wash solution. Pipette 5 ml of 100 % ethanol in 80 ml type I water in a 100 ml volumetric flask. When dissolution is complete, bring up to 100 ml with type I water and transfer to a beaker.

***Stock standard solution***

Standards are purchased as certified solutions. A fresh stock standard solution is prepared each time calibration curves are run. The 1 ppm standard is also run as a sample for quality check purposes once every 10 samples and it is stored at 4 °C between runs.

- Anions standard stock solution. Pipette 1 ml of the 1000 mg/L of the following single standard Cl ^-^, Br ^-^, NO _3_^-^, NO _2_^-^, SO _4_^2-^ and HPO _4_^2-^ in a 10 ml volumetric flask, bring up to volume with type I water.
- Cations standard stock solution. Pipette 1 ml of the 1000 mg/L of the following single standard Ca ^2+^, Na ^+^, K ^+^, Mg _2_^+^, NH _4_^+ ^in a 10 ml volumetric flask, bring up to volume with type I water.

Anions and cations standards are prepared by dilution of a mixed standard with a final concentration of 0.1, 0.2, 0.5, 1, 5, 10 mg/L of each element.

### Sample collection, preservation and storage

In the field, samples are collected and filtered through a 0.22 μm membrane filter in clean plastic tubes that are conditioned with the sample 3 times before collection. If samples are not processed immediately, they can be stored at 4 °C.

### Quality control of the analytical setup

**Figure 1.**
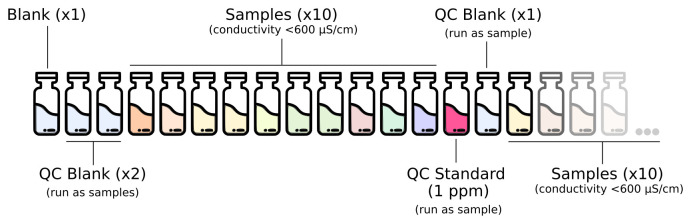
Analytical setup for the routine analysis of anions and cations in deeply-sourced seeps fluid samples.

The full analytical setup consists of a series of quality control (QC) blanks, samples and QC standard run as described in Figure 1. The QC blanks are used to verify the absence of ions carryover in the column and confirm low background levels while the QC standard is used to check for peak drift and consistent quantification of the standards.

- A full calibration is performed at least once a month, after column maintenance/replacement and in case of failed QC step while using the routine 1 ppm standard.
- Blanks composed of type I water (18 MΩ/cm) are run at the beginning of every run.
- Two QC blanks (type I water) are run as samples at the beginning of each run and once every 10 samples after the routine QC standard.
- The 1 ppm calibration standard solution is run as a sample for routine QC verification once every 10 samples.

### Analytical procedure

- Check whether the amount of eluent is sufficient for the analysis. If not, prepare the solution as described in the “Reagents and Standards” section.
- Check that the installed column is appropriate for the type of analysis. If not, disconnect the column and the guard column. Be sure to cap both ends of the guard and analytical column so they do not dry.
- Be sure that lines to pre-screen the eluent are immersed in the liquid. If switching between methods disconnect eluent lines and place the filter in a becker with type I water until the eluent is replaced to prevent the filter from drying out.
- Check that the correct loop is installed.
- Diluted each sample using type I water to a conductivity of 600 µS/cm before the analysis. 10 ml of final diluted sample is sufficient for both cations and anions analysis.
- Open the software and check that the selected method is correct.
- Click “Start HW” in the “Equilibration” windows. Allow the IC to run for 30–45 minutes until the baseline equilibrates, monitor flow, pressure and conductivity (approximately 900 μS/cm for cationic run and 15 μS/cm for anionic run).
- In “Determination series” make a sample list analysis with relative information like expedition, sample origin, sample type, position, injection loop and dilution applied to the sample.
- Organize the autosampler according to the analytical setup described in Figure 1. Mark in the software the position of the wash solution. Select “Start” to run the analysis.

### Instrument calibration

**Figure 2.**
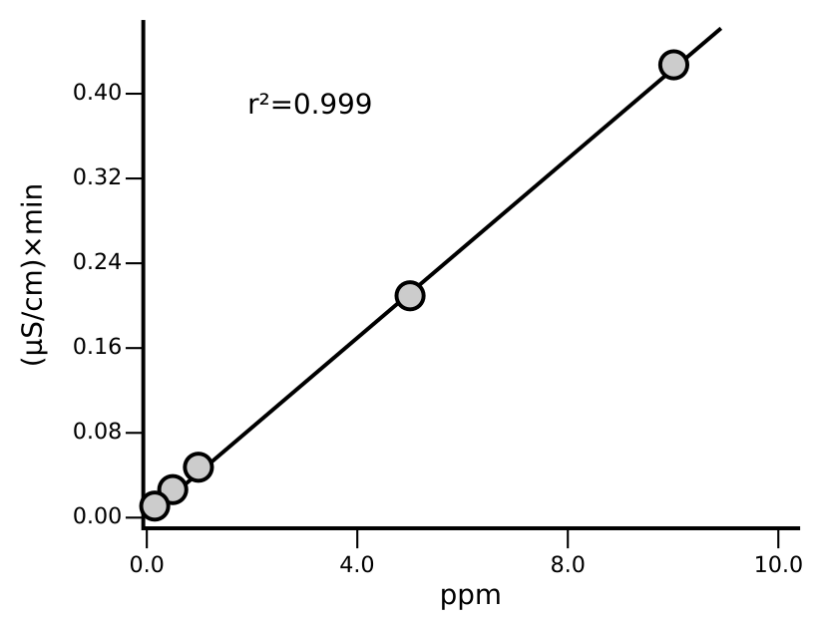
Example of calibration curves for Na ^+^ with a final concentration of 0.1, 0.2, 0.5, 1, 5, 10 mg/L determined following the described SOP.

Calibration is carried out at least once a month, after column maintenance/replacement and in case of failed QC step while using the routine 1 ppm standard. The correlation coefficient resulting from analysis is considered acceptable when r² ≥ 0.999 ( Figure 2).

***Limit of detection (LOD) and Limit of quantification (LOQ)***

Limit of detection (LOD) is the smallest measure that can be detected with reasonable certainty for an analytical procedure, while the Limit of quantification (LOQ) is the smallest concentration that can be quantified with accuracy and precision. There are several methods to calculate LOD and LOQ. We use the method based on the calibration curves ( Indrayanto, 2018).

Limit of detection (LOD) is expressed as:


LOD=3.3∗σ/S

and Limit of quantification (LOQ) is expressed as:


LOQ=10∗σ/S

Where *σ* is the standard deviation of the regression and S is the slope of the calibration curve ( Tietje & Brouder, 2010). The LOD and LOQ values obtained for our IC SOP are given in Table 1.

**Table 1.** LOD and LOQ for the IC SOP in the Giovannelli Lab at the University of Naples Federico II, Italy.

	LOD		LOQ	
Analyte	mg/L	µM	mg/L	µM
Ca ^2+^	0.0080	0.1996	0.0242	0.6038
Na ^+^	0.0104	0.4523	0.0315	1.3701
K ^+^	0.0099	0.2532	0.0300	0.7672
Mg _2_^+^	0.0056	0.2304	0.0170	0.6994
NH _4_^+^	0.0119	0.6596	0.0362	2.0066
Cl ^-^	0.0037	0.1043	0.0113	0.3187
Br ^-^	0.0566	0.7083	0.1715	2.1463
NO _3_^-^	0.0504	0.8127	0.1529	2.4657
NO _2_^-^	0.0273	0.5933	0.0829	1.8017
SO _4_^2-^	0.0190	0.1977	0.0577	0.6006
HPO _4_^2-^	0.0592	0.6168	0.1796	1.8712

## Results

### Use case

**Figure 3.**
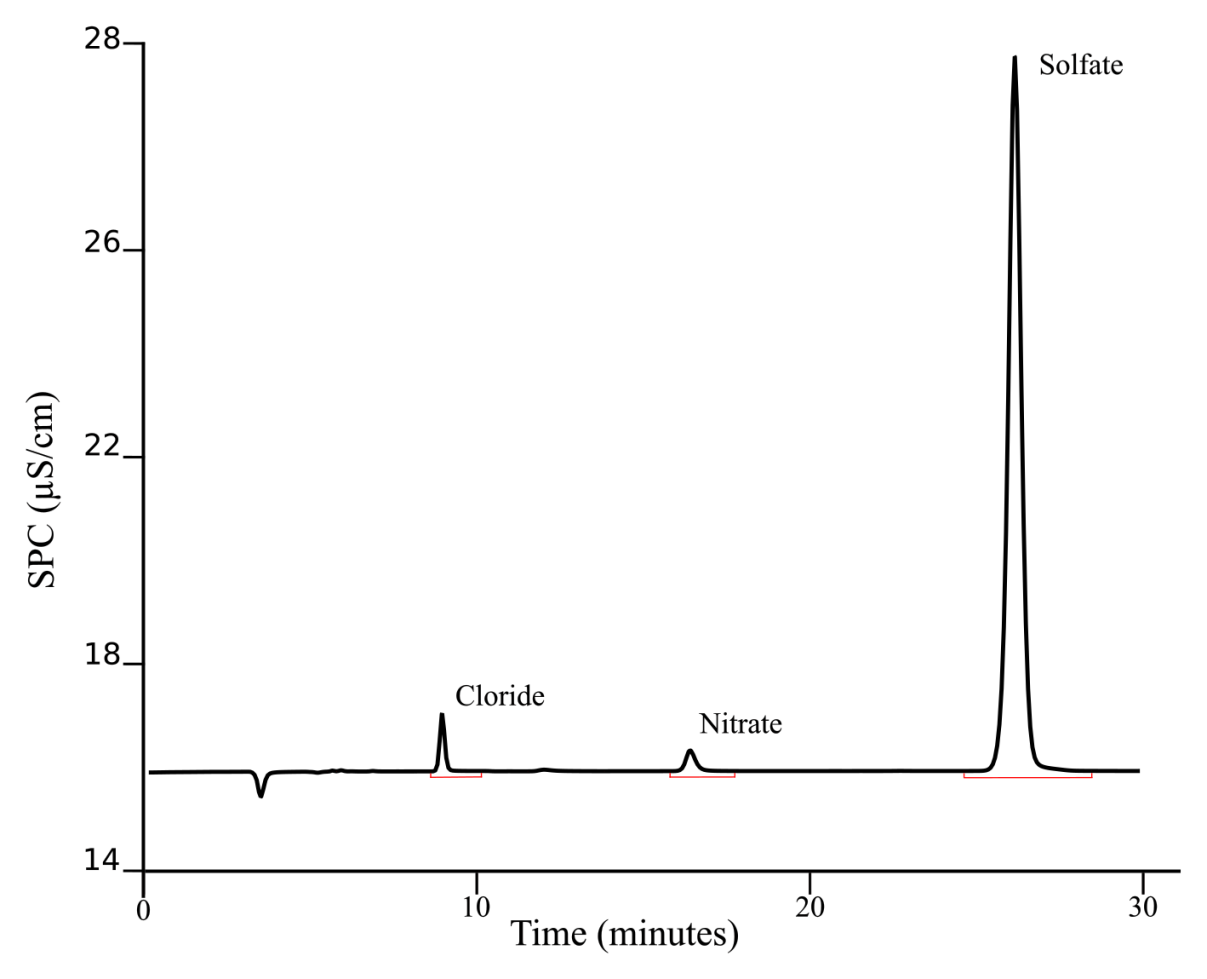
Example of chromatogram obtained from KR1 sample following the described SOP.

The proposed procedure was applied to investigate the concentration of major inorganic cations and anions in hydrothermal fluids of diverse geothermal systems, and has been successfully used in the past to investigate how the geochemical composition of the geothermal fluids influences microbial diversity ( Fullerton *et al.*, 2021). Figure 3 presents a typical chromatogram obtained after applying this procedure for separating the investigated ions in geothermal fluids.

**Figure 4.**
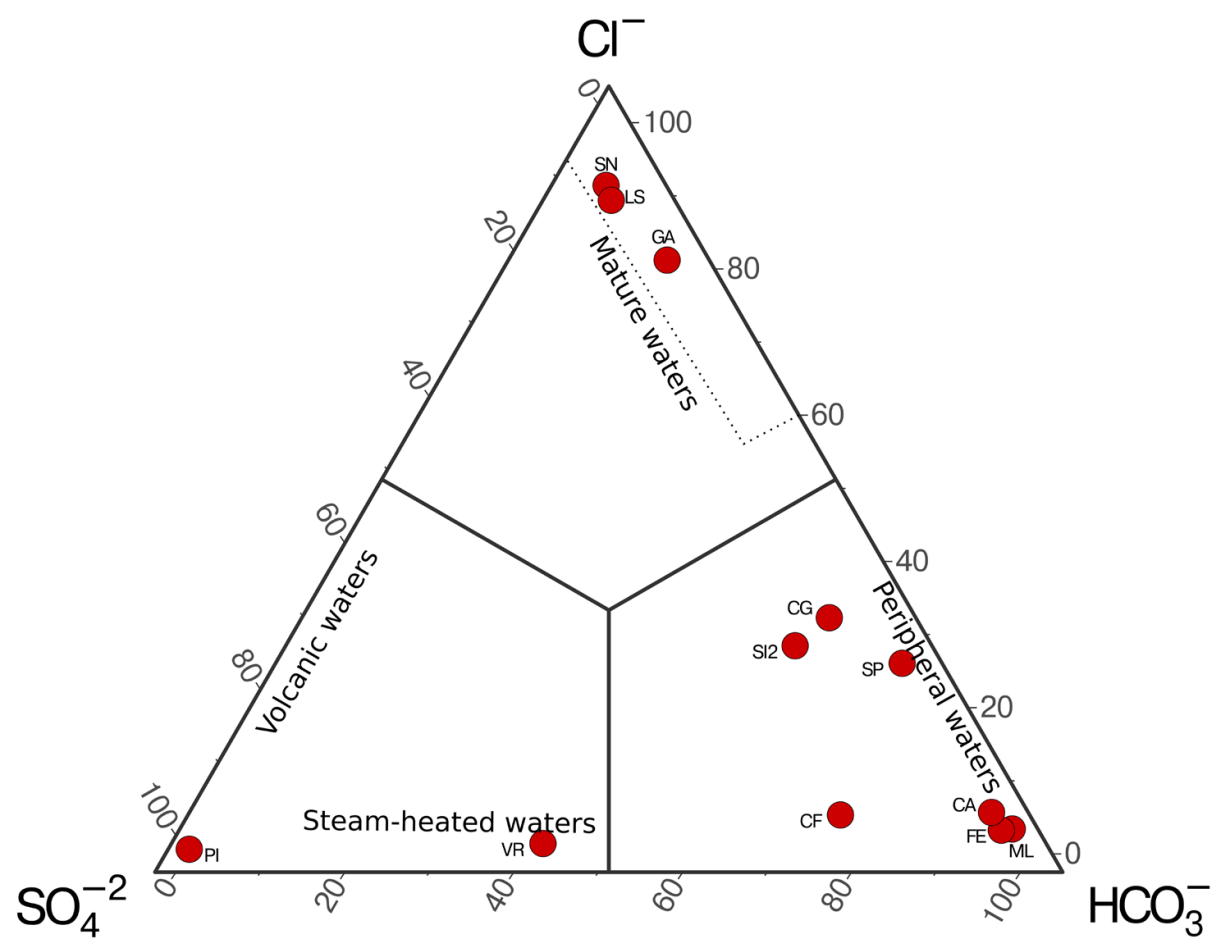
Example of ternary plot for the classification of hydrothermal fluids from the Campania Region (Italy, unpublished data) based on anion concentrations following Giggenbach (1988).

The chromatogram presented is from a sample (KR1) taken during the ICE21 expedition in Krysuvik (Iceland), a very dynamic and diverse geothermal activity region. The basic environmental parameters for this sample are reported in Table 2. The concentrations of aqueous anions (Cl ^−^, SO _4_^2−^, and HCO _3_^−^) obtained are plotted using Giggenbach’s ternary diagram ( Giggenbach, 1988), allowing us to distinguish four different types of waters: volcanic waters, steam-heated waters, mature waters, and peripheral waters ( Figure 4).

**Table 2.** Location (GPS coordinates), and physico-chemical parameters measured on the sampled locations.

Station	Latitude (°N)	Longitude (°E)	Altitude (m)	Temperature (°C)	pH	DO (%)	Spc (µS/cm)	Sal (%)
KR1	63.89546	-22.056914	246	87.5	3.1	91	2.543	0.53

A case study application of the methods was carried out using hydrothermal fluids collected throughout the Campania region (Italy). The ternary distribution of aqueous anions shows fluids from all four different types of waters. A single sample (PI) is classified as volcanic waters, and a single sample (VR) is classified as steam-heated water in spite of the low temperature of the site (12 °C at the time of sampling), which suggest a deep source for the volatile in the area that cool and mix with shallow waters during ascent. Both samples reflect water significantly affected by deep heat sources rich in sulfates. Most of the fluids are found in the peripheral water, enriched in HCO _3_^−^, due to a higher influence of meteoric waters. The remaining samples are found in the mature waters region, which are thought to represent well-equilibrated fluids from deep geothermal wells.

## Data Availability

Zenodo. giovannellilab/GiovannelliLab_SOPs: Standard Operating Procedure for the analysis of trace elements in hydrothermal fluids by Inductively Coupled Plasma Mass Spectrometry (ICP-MS). 10.5281/zenodo.7680349 ( Giovannelli, 2023) This project contains the following underlying data: Table1.csv (Contains the data for Table 1 of the manuscript.) Table2.csv (Contains the data for Table 2 of the manuscript.) SOP_IC_example_data.csv (Contains the data used in the Case Study in the Campania Region used to plot Figure 4) Data are available under the terms of the Creative Commons Attribution 4.0 International license (CC-BY 4.0).
