# Supplementary material for: Standard operating procedure for the analysis of major ions in hydrothermal fluids by ion chromatography
Source: Open Res Eur. 2024 Jul 29;3:94. Originally published 2023 Jun 14. [Version 3] doi: 10.12688/openreseurope.15605.3 (PMC11387938; doi:10.12688/openreseurope.15605.3)
Supplement: Peer review history

# Reviewer report: Reviewer response for version 3

**DOI:** 10.21956/openreseurope.19527.r43761

**Published:** 2024-09-10

**Authors:** Juris Burlakovs

**Affiliations:** 1 Riga Technical University, Riga, Latvia

the SOP for analysis of major ions is comprehensive and relevant with approved techniques and fully repeatable, results and conclusions reasonable

 the Introductory part reflects the problem and is aiming the pathway to the determination of problem and potential solutions

 methodology is sound

 the innovative pattern is obvious and is helping provide new insights in elaboration of analytical techniques

Is the rationale for developing the new method (or application) clearly explained?

Yes

Is the description of the method technically sound?

Yes

Are the conclusions about the method and its performance adequately supported by the findings presented in the article?

Yes

If any results are presented, are all the source data underlying the results available to ensure full reproducibility?

Yes

Are sufficient details provided to allow replication of the method development and its use by others?

Yes

Reviewer Expertise:

geochemistry, geology, environmental studies

I confirm that I have read this submission and believe that I have an appropriate level of expertise to confirm that it is of an acceptable scientific standard.

# Reviewer report: Reviewer response for version 3

**DOI:** 10.21956/openreseurope.19527.r42557

**Published:** 2024-09-04

**Authors:** Dzenita Avdibegovic

**Affiliations:** 1 KU Leuven, Leuven, Flanders, Belgium

The authors implemented necessary changes.

 The authors have responded and revised the SOP according to my comments.

 The SOP can be accepted.

Is the rationale for developing the new method (or application) clearly explained?

Yes

Is the description of the method technically sound?

Yes

Are the conclusions about the method and its performance adequately supported by the findings presented in the article?

Partly

If any results are presented, are all the source data underlying the results available to ensure full reproducibility?

Partly

Are sufficient details provided to allow replication of the method development and its use by others?

Partly

Reviewer Expertise:

NA

I confirm that I have read this submission and believe that I have an appropriate level of expertise to confirm that it is of an acceptable scientific standard.

# Reviewer report: Reviewer response for version 3

**DOI:** 10.21956/openreseurope.19527.r42558

**Published:** 2024-08-08

**Authors:** Łukasz Nuckowski

**Affiliations:** 1 Faculty of Oceanography and Geography, University of Gdansk (Ringgold ID: 49646), Gdańsk, Poland

1) The authors haven't shown LOD and LOQ values properly. Please express them with the same number of significant figures, not decimal places.

 2) The calibration curve needs improvement. The calibration points should be distributed evenly.

Is the rationale for developing the new method (or application) clearly explained?

No

Is the description of the method technically sound?

Yes

Are the conclusions about the method and its performance adequately supported by the findings presented in the article?

Yes

If any results are presented, are all the source data underlying the results available to ensure full reproducibility?

Yes

Are sufficient details provided to allow replication of the method development and its use by others?

Yes

Reviewer Expertise:

Chromatgoraphy

I confirm that I have read this submission and believe that I have an appropriate level of expertise to confirm that it is of an acceptable scientific standard, however I have significant reservations, as outlined above.

# Response

**Published:** 2024-08-09

**Authors:** Donato Giovannelli

**Affiliations:** Department of Biology, University of Naples Federico II, Naples, Italy

Dear Reviewer, thank you for the suggestions.

 1. I am unsure why we should drop significant digits for some of our detection limits to show the same number of significant figure for all the elements. There is no current standard that requires to drop significant digits to unify a table. So respectfully disagree on this request, and this change does not affect the validity of the SOP presented.

 2. Similarly calibration points across a calibration curve do not need to be uniformly spaced, and their distribution depends from several factors. The important aspect is covering the entire range of necessary dilutions while keeping a strong accuracy.

# Reviewer report: Reviewer response for version 1

**DOI:** 10.21956/openreseurope.16866.r40308

**Published:** 2024-05-28

**Authors:** Dzenita Avdibegovic

**Affiliations:** 1 KU Leuven, Leuven, Flanders, Belgium

The SOP is relatively clear and concise.

 However, I recommend to consider and include the following corrections:

- The authors recommend to store the samples at  under the condition that they are "not processed" immediately. The word "processed" is not very clear. Thus, I advise that the authors recommend to store the samples at 4 °C until they are ready to be prepared for analysis.
- The authors should include for how long the samples can be stored at 4 °C, as well as to add recommendations in case samples cannot be analyzed during that time (e.g. in case of equipment failure - is freezing recommended in that case?
- In case the samples are filtered prior to analysis it is advisable that the blanks are filtered as well
- The authors should recommend standards/sample injection volume
- The authors should give recommendations on number of replicates
- The authors should give recommendations on spiked samples
- The authors should advise on data interpretation in case the results fall in the region between LOD and LOQ
- The authors should add a QC sample after blank, thus at the beginning of the sample sequence
- The authors should include the criteria for QC (recovery %) and correction actions in case of QC failure
- The authors should add recommendations to prepare series of calibration standards in the low region, middle region and high region

Is the rationale for developing the new method (or application) clearly explained?

Yes

Is the description of the method technically sound?

Yes

Are the conclusions about the method and its performance adequately supported by the findings presented in the article?

Partly

If any results are presented, are all the source data underlying the results available to ensure full reproducibility?

Partly

Are sufficient details provided to allow replication of the method development and its use by others?

Partly

Reviewer Expertise:

Analytical chemistry, ion exchange, chromatography

I confirm that I have read this submission and believe that I have an appropriate level of expertise to confirm that it is of an acceptable scientific standard, however I have significant reservations, as outlined above.

# Response

**Published:** 2024-06-12

**Authors:** Donato Giovannelli

**Affiliations:** Department of Biology, University of Naples Federico II, Naples, Italy

We thank the reviewer for the useful comments. We respond point by point below.

 1. Thank you for pointing this out! We routinely store at +4˚C in the dark. We have corrected these confusing sentences (pag 7).

 2. We have added a brief description of sample preservation and citation that support this sentence (pag 7).

 3. The leachable ions are very labile and are essentially removed completely if the filter is pre-washed with water or conditioned with the sample before collection, as we do in the field. That said, filter and reagent blanks are routinely analyzed as part of the QC in the lab.  We have now explained the conditioning of the membrane filter in the text (pag 7).

 4. We have added the loop injection volume. (pag 8)

 5. We have added a brief sentence mentioning the replicates.(pag 10) 

 6. We have added a brief sentence mentioning the standard addition method. (pag 7)

 7. In environmental samples it is rare that the results falls in the region between LOD and LOQ, but we have added a brief sentence mentioning this point (pag 9)

 8. Considering that at the end of each set of samples, and thus also before the end of the analysis, we run a standard and a blank, we do not deem it necessary to run another standard before the start of the analysis.

 9. We have added a brief sentence mentioning the QC failure in QC section (pag 7)

 10. We have added a brief sentence mentioning the calibration standard point. (pag 6)

# Reviewer report: Reviewer response for version 1

**DOI:** 10.21956/openreseurope.16866.r35916

**Published:** 2023-11-30

**Authors:** Łukasz Nuckowski

**Affiliations:** 1 Faculty of Oceanography and Geography, University of Gdansk (Ringgold ID: 49646), Gdańsk, Poland

The authors do a lot of work preparing the manuscript. They provided all the data necessary to repeat and apply the described method.

 However, I do not see any differences between the given method and the standard method described by IE column producers without any changes.

 Moreover, I have a few minor comments on the article:

 1) The authors describe that samples were stored at 4 °C in the dark until analysis. Is there any data to prove their stability?

 2) Calibration curve in Figure 2. is poor. It shows only 3 regularly distributed calibration points. In this way, the correlation coefficient will always be high.

 3) LOD and LOQ are displayed with different numbers of significant places. Their number should be standardized, for example, by using exponential notation.

Is the rationale for developing the new method (or application) clearly explained?

No

Is the description of the method technically sound?

Yes

Are the conclusions about the method and its performance adequately supported by the findings presented in the article?

Yes

If any results are presented, are all the source data underlying the results available to ensure full reproducibility?

Yes

Are sufficient details provided to allow replication of the method development and its use by others?

Yes

Reviewer Expertise:

Chromatgoraphy

I confirm that I have read this submission and believe that I have an appropriate level of expertise to confirm that it is of an acceptable scientific standard, however I have significant reservations, as outlined above.

# Response

**Published:** 2024-04-30

**Authors:** Donato Giovannelli

**Affiliations:** Department of Biology, University of Naples Federico II, Naples, Italy

We want to thank the reviewer for the useful comments. We respond point by point below.   1. We follow the procedure for sample handling and preservation reported in the Standard Methods for the analysis of waters and wastewater Ed. 23, method 4110 B. Filtration is carried out at 0.22 µm instead of 0.45 µm as reported in the 4110 B method to remove microorganisms and stored at +4 °C.  

 2. The reported calibration in Figure 2 contains 6 points with concentration of 0.1, 0.2, 0.5, 1,     5, 10 mg/L, as visible in the graph and reported in the caption.  

 3. Al the LOD and LOQ are reported at the 4th significant place.
